# Schizophrenia and Orthoptic Conditions: A Literature Review

**DOI:** 10.22599/bioj.327

**Published:** 2024-04-26

**Authors:** Anna McBride, Gemma Arblaster

**Affiliations:** 1 University of Sheffield (BMedSci Orthoptics), UK; 2 University Hospitals Dorset, UK; 3 School of Allied Health Professions, Nursing and Midwifery, Faculty of Health, University of Sheffield, UK

**Keywords:** schizophrenia, anti-psychotic medication, strabismus, smooth pursuit, prosaccade, stereoacuity

## Abstract

**Purpose:**

A narrative review of the literature reporting ocular abnormalities in patients with schizophrenia was undertaken to determine the types and prevalence of orthoptic conditions in this patient cohort.

**Methods:**

A systematic search of multiple databases yielded 1,974 studies published between January 1992 and January 2022. All were screened for relevance based on their title and abstract.

**Results:**

Seventeen studies were included in the final review. Ocular abnormalities reported in schizophrenia included a high incidence of strabismus, reduced visual acuity and reduced stereopsis compared to controls. Additionally, eye movement abnormalities (including reduced smooth pursuit gain and increased prosaccade latency) were frequently reported. Reduced visual acuity was associated with negative symptoms and reduced quality of life in schizophrenia.

**Conclusions:**

Orthoptists and eye care professionals should be aware that a higher incidence of strabismus, reduced visual acuity, reduced stereoacuity, and eye movement abnormalities are reported in patients with schizophrenia. Further research is required to determine whether, or to what extent, ocular abnormalities and visual disturbances influence or exacerbate the symptoms of schizophrenia, and whether there is an effect of schizophrenia medication on these orthoptic conditions.

## Background

Schizophrenia is a psychiatric disorder affecting approximately 1% of the global population (Császár, Kapocs & Bokkon 2019). Patients with schizophrenia typically present with distorted thinking, perception, mood or behaviour, commonly experiencing visual and/or auditory hallucinations (World Health Organization International Classification of Disease (1CD-10) 2019). These symptoms can be further categorised as ‘negative’, ‘positive’, and ‘cognitive’. Negative symptoms involve diminished responsiveness, social interactions, and physical activity. Positive symptoms are hallucinations and delusions, and cognitive symptoms involve issues with information retention and memory (Jones & Buckley 2006). The aetiology of schizophrenia is not well understood; both genetic and environmental factors are considered contributory (Jablensky 2010). An imbalance of neurotransmitters dopamine, glutamate, and serotonin is commonly reported (Császár, Kapocs & Bokkon 2019) and structural changes to the brain are observed, including a reduction in grey matter, reduced cortical thickness, and larger lateral ventricles than ‘controls’ (Jones & Buckley 2006). Potential causes in utero have also been explored, such as perinatal toxoplasmosis exposure (Torrey & Yolken 2017). Additionally, environmental factors such as cannabis use (Vilain et al. 2013) have been linked to schizophrenia, perhaps prompting illness onset in the genetically vulnerable.

Prompt initiation of drug therapy in acute episodes of schizophrenia is recommended due to the rapid onset of structural neurological changes, such as progressive ventricle enlargement (Patel et al. 2014). In acute-psychotic episodes, drug therapy is used to regain ‘normal functioning’, followed by long-term maintenance therapy to combat negative symptoms. Anti-psychotic drugs offered to patients with schizophrenia alter neurotransmitter levels. These may be ‘first generation’ (‘typical’ antipsychotics work via blocking dopamine receptors) or ‘second generation’ (‘atypical’ anti-psychotics block dopamine receptors and influence serotonin levels) (Vanasse et al. 2016).

Visual distortions and ocular abnormalities have been reported in patients with schizophrenia, including inaccurate and poorly controlled saccades and smooth pursuit (Jurišić et al. 2020), higher incidence of strabismus, and increased rates of reduced visual acuity than in ‘healthy controls’ (Silverstein & Rosen 2015). The lack of reported schizophrenia in congenitally blind patients also suggested an association between ocular abnormalities and schizophrenia. This is explored by Landgraf and Osterheider (2013) in their ‘Protection-against-Schizophrenia’ (PaSZ) model, which suggested that deteriorated visual input contributes to the development of schizophrenia. It is also possible that anti-psychotic medication and health comorbidity in patients with schizophrenia may account for some of the ocular changes reported and symptoms experienced.

The aim of this literature review was to establish if any ocular conditions commonly seen by an orthoptist were more common in patients with schizophrenia.

## Methods

A systematic search of the published literature was performed using the following databases: Medine via Ovid SP, The Cochrane Library, Web of Science, Scopus, and The British and Irish Orthoptic Journal. The search was limited to publications in English from January 1992 to January 2022. Publications predating 1992 were not included due to the International Classification of Diseases (ICD-10) updating in 1992 to include more specific diagnostic criteria for schizophrenia (Jablensky 2010).

The search terms used are shown in Table 1.

**Table 1:** Search string used to retrieve literature.


POPULATION	EXPOSURE	OUTCOME

1. “schizophreni*”	2. “medication” 3. “ophthalmology” 4. “orthoptics” 5. “optometry” 6. “vision” 7. “eye test” 8. “visual” 9. “vision test*”	10. “Strabismus” 11. “squint” 12. “stereo*” 13. “eye*” 14. “eye movements” 15. “ocular” 16. “binocular Vision”

	Combine terms 2 to 9 using ‘OR’	Combine terms 10 to 16 using ‘OR’

Combine 1, 9, and 16 using ‘AND’		


**Figure 1:**
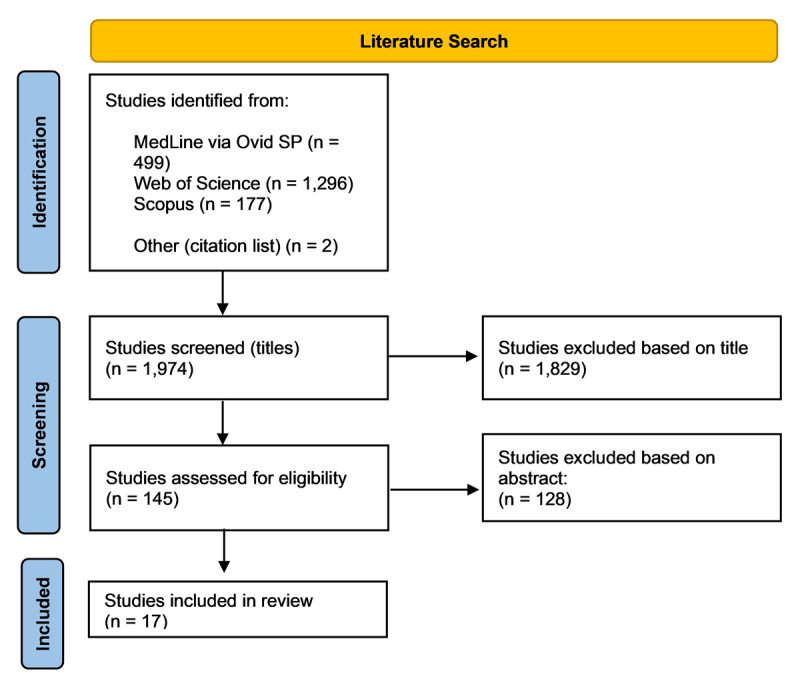
PRISMA flow chart showing the literature search results.

Following the initial search, duplicates were removed. The titles and abstracts of remaining papers were then screened for relevance to the research question, and those deemed not relevant were discarded. All remaining articles were read in full to determine suitability for inclusion. The reference lists of relevant articles were also searched manually to identify possible studies that had not been yielded from the database search; two additional studies were selected for inclusion. The PRISMA flow diagram (Page et al. 2021) illustrating the search results, is shown in Figure 1.

## Results

Seventeen studies were included in the final literature review. Themes identified include visual acuity and refractive error (n = 5), strabismus (n = 3), eye movement abnormalities (n = 6), and stereopsis (n = 3). The following tables (2–5) summarise the number of participants, research design (Centre for Evidence-Based Medicine 2022), tests performed, and findings for each of the studies included. A summary of the evidence reporting visual acuity and refractive error is shown in Table 2.

**Table 2:** Studies reporting the prevalence and types of visual acuity and refractive error abnormalities in patients with schizophrenia.


AUTHOR(S) & TITLE	NUMBER OF PARTICIPANTS	RESEARCH DESIGN	TESTS PERFORMED	FINDINGS

Kraehenmann, R. et al. (2012). ‘Crowding deficits in the visual periphery of schizophrenia patients’	Schizophrenia (n = 20) and ‘healthy’ controls (n = 20)	Observational case-control study	Identification of optotypes (binocularly) presented 0.75 m away with crowding in the periphery (own test designed for study)	Patients with schizophrenia were less accurate identifying letters with crowding than healthy controls (p = 0.003), performing more accurately when letters were spaced apart

Cumurcu, T. et al. (2015). ‘Refraction and eye anterior segment parameters in schizophrenic patients’	Schizophrenia (n = 70) and ‘healthy’ controls (n = 60).	Observational case-control study	Axial length and lens thickness measured using biometry, anterior chamber assessed using Scheimpflug camera. Patients with schizophrenia completed three questionnaires relating to symptoms.	No difference in refractive error (p = 0.082) between patients with schizophrenia and controls, greater lens thickness in schizophrenia (p = 0.006), and a correlation between astigmatism and negative symptoms in schizophrenia (p = 0.008)

Zheng, W. et al. (2015). ‘Frequency and correlates of distant visual impairment in patients with schizophrenia, bipolar disorder, and major depressive disorder’	Adult psychiatric inpatients with schizophrenia, bipolar disorder, and major depressive disorder (n = 356)	Cohort study	Vision assessed using a LogMAR chart (2.5 meters, binocularly), symptoms assessed using Brief Psychiatric Rating Scale (BPRS). Quality of life assessed using Chinese version of 12-item Short-Form Medical Outcomes Study (SF-12)	15.2% of patients with schizophrenia had distance visual impairment, similar to range reported in Chinese general population (5.4%–15.8%). Patients with distance visual impairment had lower quality of life scores (SF-12).

Hayes, J. F. et al. (2019). ‘Visual acuity in late adolescence and future psychosis risk in a cohort of 1 million men’	Swedish military conscripts (male only) (n = 1,140,710).	Longitudinal cohort study	Distance visual acuity assessed by an Optometrist using Snellen (from January 1974 to December 1997). Data extracted from the ‘National Patient Register’ (across same time period) regarding in-patient treatment for ‘psychosis’.	20.82% of cohort had refractive error, 1.1% had reduced vision which could not be corrected. Impaired VA associated with increased incidence of schizophrenia diagnosis (adjusted HR = 1.31) (95% CI = 1.22–1.41), and higher general psychotic illness rates. Anisometropia associated with increased incidence of psychosis.

Shoham, N. et al. (2021). ‘Association Between Childhood Visual Acuity and Late Adolescent Psychotic Experiences: A Prospective Birth Cohort Study’	Individuals from the Avon Longitudinal Study of Parents and Children (ALSPAC) (n = 6686)	Longitudinal cohort study	Orthoptic assessment at ages 7 and 11, then completed the ‘Psychotic-Like Screening Symptoms Interview’ (PLIKSi) at ages 17 and 24.	Odds of psychotic experience in adulthood increased with every 0.1 reduction in VA (LogMAR) at age 7. A positive result on PLIKSi more likely for those who wore glasses at age 7 (p = 0.39) or age 11 (p = <0.01).


Confidence interval (CI) describes the chance that the reported range excludes the mean of the population (Swinscow 2002). Hazard ratio (HR) the ratio of the hazard rate in the control group in comparison to the hazard rate in the exposed group (Spruance et al. 2004).

A summary of the evidence reporting eye movement abnormalities, including smooth pursuit, saccades (prosaccades and antisaccades), convergence, fusional vergence, and eye movements in ‘natural’ environments, is seen in Table 3.

**Table 3:** Studies reporting the prevalence and type of eye movement abnormalities in patients with schizophrenia.


AUTHOR(S) & TITLE	NUMBER OF PARTICIPANTS	RESEARCH DESIGN	TESTS PERFORMED	FINDINGS

Friedman et al. (1992). ‘Effect of typical antipsychotic medications and clozapine on smooth pursuit performance in patients with schizophrenia’	Schizophrenia (n = 13) and ‘healthy’ controls (n = 19)	Repeated measures experiment	Smooth pursuit movements recorded monocularly using infrared oculography for three separate groups. Group 1 (schizophrenia) tested drug-naïve and then taking typical antipsychotics; group 2 (schizophrenia) tested drug-naïve and then taking Clozapine; group 3 were drug free non-schizophrenic controls.	Unmedicated schizophrenia patients had low smooth pursuit gain (p = 0.004) and more catch-up saccades than controls (p = 0.025). Patients taking Clozapine had highest amplitude of catch-up saccades across three groups (p = 0.0277). Typical antipsychotics did not significantly influence smooth pursuit.

Sereno, A. B., Holzman, P. S. (1995). ‘Antisaccades and smooth pursuit eye movements in schizophrenia’	Schizophrenia (n = 17), bipolar (n = 11), and ‘healthy’ controls (n = 14)	Observational case-control study	All three groups were given a prosaccade, antisaccade, and smooth pursuit task, measured using ISCAN infrared eye tracking system.	Controls demonstrated normal smooth pursuit. 10/15 patients with schizophrenia had impaired smooth pursuit. Patients with schizophrenia had increased antisaccade error rate (p = <0.0005). Impaired smooth pursuit correlated with prosaccade latency (not statistically significant (p = >0.12)).

Hutton et al. (2001). ‘Short- and long-term effects of antipsychotic medication on smooth pursuit eye tracking in schizophrenia’	Chronic schizophrenia, (n = 36), first-episode schizophrenia (n = 67), and ‘healthy’ controls (n = 54)	Observational case-control study	Chronic and first-episode schizophrenia patients split into groups: drug-naïve (n = 20 first-episode, n = 16 chronic) and medicated (n = 47 first-episode, n = 20 chronic). Smooth pursuit movements recorded using infrared eye tracking system.	Smooth pursuit gain reduced in chronic (p = <0.001) and first-episode (p = <0.001) patients compared to controls, chronic patients most significantly impaired. Smooth pursuit of antipsychotic naïve and antipsychotic treated first episode patients not different (p = 0.37). Antipsychotic treated chronic patients had more reduced gain than antipsychotic free chronic patients (p = >0.005).

Bolding, M.S. et al. (2012). ‘Ocular convergence deficits in schizophrenia’	Schizophrenia (n = 20) and ‘healthy’ controls (n = 20)	Observational case-control study	Near point of convergence measured three times using Astron International Accommodative Rule. Positive fusional vergence measured three times using prism bar. Participants completed the Convergence Insufficiency Symptom Survey (CISS).	Mean near point of convergence 5.5 cm for patients with schizophrenia and 4.4 cm for controls, not significantly different (p = 0.207). Mean CISS score for patients with schizophrenia higher than for controls (18.6 ± 9.9, compared to 9.9 ± 5.5). Patients with schizophrenia more likely to fail Sheard’s criteria (p = 0.022).

Dowiasch et al. (2016). ‘Eye movements of patients with schizophrenia in a natural environment’	Schizophrenia (n = 20) and ‘healthy’ controls (n = 20)	Observational case control study	EyeSeeCam (ESC) tracking device used to record eye movements whilst completing four separate oculomotor tasks: fixating on a pre-defined target, free gaze whilst sat in a hallway, free gaze whilst walking, and tracking a fixed spot whilst walking towards it.	No significant difference in gain during spontaneous tracking(p = 0.617) or tracking a fixed spot (p = 0.654). Self-initiated saccades showed significant ‘undershoots’ in schizophrenia, but saccade amplitude and frequency not significantly different from controls.

Subramaniam et al. (2018). ‘Clinical correlates of saccadic eye movement in antipsychotic-naive schizophrenia’	Schizophrenia (n = 45) and ‘healthy’ controls (n = 57)	Observational case-control study	EyeLink 2000 recorded eye movements whilst patients fixated on 0.3 cm circle displayed on monitor. Participants performed 24 prosaccade tasks and 48 antisaccade tasks.	Patients with schizophrenia significantly more likely to make antisaccade and final eye position errors in comparison to controls (p = <0.001). Latency of correct prosaccades greater for patients with schizophrenia (p = 0.003) and amplitude gain of correct prosaccades reduced (p = 0.03).


A prosaccade is a saccade made in the same direction as a peripheral target to refixate the fovea on to the target. An antisaccade is a saccade made in the opposite direction to a peripheral target, demonstrating inhibitory control (Magnusdottir et al. 2019).

A summary of the evidence reporting prevalence and types of strabismus is shown in Table 4.

**Table 4:** Studies exploring the prevalence of strabismus in patients with schizophrenia.


AUTHOR(S) & TITLE	NUMBER OF PARTICIPANTS	RESEARCH DESIGN	TESTS PERFORMED	FINDINGS

Toyota et al. (2004). ‘Association between schizophrenia with ocular misalignment and polyalanine length variation in PMX2B’	Schizophrenia (n = 346) and ‘healthy’ controls(n = 542)	Observational case-control study	Participants underwent Hirschberg test, cover-uncover test, and alternating cover test, performed by medical doctors (reviewed by ophthalmologist). Also screened for mutations in candidate gene PMX2B in 24 patients with schizophrenia.	Strabismus highly associated with schizophrenia in comparison to controls (p = <0.0001), particularly constant exotropia (p = <0.0001). 5% of control group had strabismus, 13% of patients with schizophrenia had strabismus.

Schiffman et al. (2006). ‘Premorbid childhood ocular alignment abnormalities and adult schizophrenia-spectrum disorder’	265 children (242 followed up into adulthood)	Longitudinal cohort study	Cohort split in to three groups: parent(s) with schizophrenia (n = 90), parent(s) with a different psychiatric condition (n = 93), and parents without psychiatric diagnosis (n = 82). Participants underwent an ‘eye examination’ by a neurologist at age 11 (in 1972). Adult psychiatric outcome data obtained in 1991.	No statistically significant association between genetic risk for schizophrenia and strabismus aged 11(p = 0.301). Children who had strabismus (or a higher ‘eye examination score’) more likely to develop a schizophrenia-spectrum disorder in later life (p = 0.33), however did not reach statistical significance.

Yoshitsugu et al. (2006). ‘A novel scale including strabismus and ‘cuspidal ear’ for distinguishing schizophrenia patients from controls using minor physical anomalies’	Schizophrenia (n = 475) and ‘healthy’ controls(n = 697)	Observational case-control study	Participants scanned for 14 minor physical anomalies (MPAs) (including strabismus) by doctor, 16 patients with schizophrenia co-examined to assess reliability. Logistic regression analysis used to identify which MPAs could be most accurately discriminated between patients and controls.	Greater frequency of strabismus within schizophrenia patient group (13%) than controls (4.4%) (p = 0.000000104), constant and intermittent exotropia over-represented. ‘Six-item’ scale of MPAs (including strabismus) could correctly classify schizophrenia in 59.6% of patients (78.9% controls correctly identified).


A summary of the research design and reports of studies reporting on stereopsis and/or depth perception abnormalities is shown in Table 5.

**Table 5:** Studies exploring stereopsis and/or depth perception deficits in patients with schizophrenia.


AUTHOR(S) & TITLE	NUMBER OF PARTICIPANTS	RESEARCH DESIGN	TESTS PERFORMED	FINDINGS

Schechter et al. (2006). ‘A new dimension of sensory dysfunction: stereopsis deficits in schizophrenia’	Schizophrenia (n = 17) and ‘healthy’ controls (n = 19)	Observational case-control study	Graded Circles Stereo Test to assess stereoacuity. BPRS to assess negative symptoms in patients with schizophrenia.	Patients with schizophrenia had reduced stereoacuity (mean = 142 seconds of arc) compared to controls (55 seconds of arc) (p = 0.006). No significant correlations between stereopsis and negative symptoms or antipsychotic dose.

Barbato et al. (2014). ‘Binocular depth perception in individuals at clinical high risk for psychosis: No evidence of dysfunction	42 young adults at ‘clinical high risk’ of developing psychosis (n = 42), and ‘healthy’ controls (n = 44)	Observational case-control study	Computerised Binocular Depth Perception test (BDP) wearing 3D ‘shutter’ glasses, judging the relative depth of two targets (own test designed for study). Subgroup (14 patients with schizophrenia, 14 controls) also undertook Stereo Butterfly Test.	Depth perception not impaired in high clinical risk group; they performed similarly to the control group on the computerised BDP test and Stereo Butterfly Test (both p = > 0.5).

Hui et al. (2017). ‘Stereopsis deficits in patients with schizophrenia in a Han Chinese population’.	Strabismus (n = 100) and non-strabismic controls (n = 80)	Observational case-control study	Titmus Stereopsis Test. Patients with schizophrenia assessed by psychiatrist using Chinese versions of Scales for Assessment of Positive and Negative Symptoms.	Stereoacuity significantly reduced in patients with schizophrenia compared to controls (p = <0.0001) (60” arc, compared to 40”arc). Stereoacuity not associated with symptom severity.


## Discussion

This systematic search of the literature has identified several visual and ocular abnormalities in people with schizophrenia. Overwhelmingly, observational case control studies have been used to investigate the ocular characteristics of schizophrenia in comparison to controls. Randomised trials are ethically impossible in this cohort, and the review therefore relies on non-randomised studies of intervention. The reliability and clinical significance of these findings will be discussed.

### Visual acuity

Two longitudinal cohort studies supported an association between either (or both) refractive error or reduced visual acuity in childhood/early adulthood and a later diagnosis of schizophrenia. Hayes et al (2019) reported that reduced visual acuity aged 18–19 was associated with a higher incidence of schizophrenia diagnosis in later life (see Table 2). When reduced visual acuity could be improved to ‘normal’ levels with glasses alone, an association with schizophrenia was still identified. Similarly, Shoham et al. (2021) found a positive correlation between reduced visual acuity in childhood and likelihood of experiencing psychosis (including schizophrenia) in later life. In addition, children who wore glasses at age 11 were significantly more likely to exhibit ‘psychotic-like’ symptoms as young adults (see Table 2), however they were unable to comment on compliance with glasses wear (due to the large retrospective cohort study design). These findings were consistent with the PaSZ (Landgraf & Osterheider 2013), in which both ‘absent’ and ‘perfect’ vision appeared protective against developing schizophrenia.

Not all sources of evidence associated reduced vision with schizophrenia. Zheng et al. (2015) reported the prevalence of distance visual impairment in in-patients with schizophrenia (15.2%) was similar to the general Chinese population (ranging from 5.4%–15.8%). Visual acuity was measured corrected only in those who had taken their glasses into hospital. All others had visual acuity measured uncorrected. Whilst this made visual acuity less standardised, it was arguably more representative of ‘real life’ viewing conditions for patients. This study (Zheng et al. 2015) had several limitations that undermined its clinical application. It was unclear whether visual acuity was tested binocularly or uniocularly. No conclusions could therefore be made about anisometropia and/or amblyopia. Furthermore, visual acuity of 0.5 LogMAR or worse was considered ‘reduced’, however visual acuities ‘greater’ than this (e.g., between 0.3 and 0.5 LogMAR) may still be considered reduced compared to normal vision (–0.16 LogMAR for healthy adults) (Elliott, Young & Whittaker 1995).

Cumurcu et al.’s (2015) case-control study found that refractive error rates did not differ between patients with schizophrenia and controls (see Table 2). Those with schizophrenia did, however, exhibit structural ocular differences, with increased lens thickness and corneal astigmatism (see Table 2). Only participants who had been taking anti-psychotic medicine for at least two years were included in this study. Therefore, it is not possible to determine whether ocular structural changes preceded or followed antipsychotic drug therapy. Furthermore, patients with amblyopia were excluded from the study, meaning the study was unable to draw conclusions regarding the relationship between amblyopia and schizophrenia.

Whilst Hayes et al. (2019) reported anisometropia was associated with an increased risk of psychosis, their analysis compared interocular difference in visual acuity. The hazard ratio for psychosis increased from 1 (no interocular difference) to 1.37 (0.30 LogMAR interocular difference). It was unclear whether those grouped as ‘anisometropic’ were amblyopic or had equal vision, leaving it difficult to draw firm conclusions on any associations between anisometropia or amblyopia (or both) and psychosis. Kraehenhamm et al.’s (2012) study of ‘crowding’ in the visual periphery suggested that patients with schizophrenia were more impacted by ‘crowding’ than controls. Testing was binocular and it was unclear whether any study participants had amblyopia and/or reduced vision. The clinical implications of these findings of crowding are therefore unclear.

Clear, equal vision facilitates independence, aids social interaction, and thus encourages a higher quality of life than for those with sight impairment. Stelmack (2001) found that patients with visual impairment were at greater risk of depression, and depression has been linked to poor functional outcomes in schizophrenia (Conley et al., 2007). This trend was also highlighted in studies examining visual acuity deficits in patients with schizophrenia. Zheng et al. (2015) found that in-patients with distance visual impairment (visual acuity worse than 0.5 LogMAR) rated their quality of life in relation to ‘general vision’ as significantly lower than those without visual impairment, and these patients also reported more difficulty with ‘social functioning’. Additionally, Cumurcu et al. (2015) reported a correlation between astigmatism and increased negative symptoms of schizophrenia (see Table 2), supporting the premise that refractive error and/or visual acuity deficits may contribute to social withdrawal in this subgroup. Considering this impact, regular eye examinations are of increased importance for patients with schizophrenia to minimise the potential impact of visual acuity deficits on daily functioning.

Hayes et al.’s (2019) and Shoham et al.’s (2021) longitudinal cohort studies included a large cohort of participants, supporting the reliability of their findings. However, Hayes et al.’s (2019) cohort included only male military conscripts, and it is unclear whether findings would be replicated across a female population. Furthermore, the findings from Shoham et al.’s (2021) almost entirely white, British cohort of participants perhaps cannot be extrapolated to that of the general population, and it is unclear whether findings differ across ethnicities. Future research should include a wider range of participants, both male and female, to conclude whether findings can be generalised across different populations.

There appears to be a consensus that visual acuity deficits and schizophrenia are associated, however further research is required to make more definite conclusions about this relationship, and to develop considerations regarding the impacts of drug therapy and amblyopia. Current research also highlights the impact reduced vision has on quality of life for patients with schizophrenia, suggesting that orthoptic or ophthalmological intervention could contribute to holistically treating negative symptoms. However, whilst some studies have identified an association between reduced visual acuity (and/or refractive error) and increased incidence of schizophrenia (Hayes et al. 2019; Kraehenmann et al. 2012; Shoham et al. 2021), it is unclear whether this is correlation or causational.

### Eye movements

Eye movement abnormalities are considered important biomarkers across a range of neurological conditions (Hashimoto 2021), including Parkinson’s disease and multiple sclerosis. Research has also highlighted an association between eye movement abnormalities and schizophrenia, offering an insight into the neurological characteristics of this condition.

#### Smooth pursuit

There is a well-established association between smooth pursuit abnormality and schizophrenia. Friedman et al.’s (1992) study found that unmedicated patients with schizophrenia had reduced smooth pursuit gain and made more catch-up saccades in comparison to ‘healthy’ controls (see Table 3). These findings were supported by Hutton et al. (2001), who found that smooth pursuit gain was reduced in both first episode and chronic schizophrenia patients (see Table 3), across both medicated and unmedicated groups. These findings suggest that reduced smooth pursuit gain is intrinsic to schizophrenia itself, not a side effect of anti-psychotic medication. These findings were also highlighted by Sereno and Holzman (1995), who found that 10/15 patients with schizophrenia had ‘abnormal’ smooth pursuit (however the exact mechanisms of ‘abnormality’ were not explored). The artificial, lab-based environments of these studies may have influenced findings. Dowiasch et al. (2016) suggested that the smooth pursuit abnormalities repeatedly identified in lab-based studies may not be representative of real-life viewing conditions; they found no significant difference in smooth pursuit gain during spontaneous tracking of a fixed spot (see Table 3). This may be due to head movements ‘compensating’ for any smooth pursuit deficits, which is not possible during lab experiments using a chin rest.

Studies have also explored the impact of long-term antipsychotic treatment on smooth pursuit in patients with schizophrenia. Chronic patients (those with long term negative symptoms (ICD-10 2019)) treated with typical antipsychotics had more significantly impaired smooth pursuit than unmedicated chronic patients in Hutton et al.’s (2001) study (see Table 3), demonstrating reduced velocity gain. Additionally, Friedman et al. (1992) found that patients medicated with Clozapine (an ‘atypical’ antipsychotic) showed significantly more catch-up saccades during smooth pursuit than those taking ‘typical’ antipsychotics (see Table 3), perhaps due to Clozapine’s sedative effects (Hutton et al., 2001). Friedman et al. (1992) also found that age and illness duration (related variables) positively correlated with improvement of smooth pursuit gain in medicated patients, whereas Hutton et al. (2001) found chronic, medicated patients had the most impaired smooth pursuit. These discrepancies of findings could be due to the nature of patients in Hutton et al.’s (2001) ‘unmedicated’ cohort; potentially, chronic patients with less severe symptoms were more likely to remain unmedicated, which could be a confounding variable, accounting for the less impaired smooth pursuit in this subgroup. The relationship between schizophrenia symptom severity and reduced smooth pursuit gain remains unclear, and further research is required. Also, the small sample size of Friedman et al.’s (1992) study could lead us to question the validity of their findings, along with the short duration that the ‘unmedicated’ subgroup had been drug free – a minimum of eight days, in comparison to a minimum of six months for Hutton et al.’s (2001) ‘unmedicated’ cohort.

#### Prosaccades

An increased latency of prosaccades in patients with schizophrenia has been reported. Sereno and Holzman (1995) found that patients with schizophrenia who demonstrated impaired smooth pursuit also demonstrated increased saccadic latency (see Table 3); however, this finding did not reach statistical significance and definite conclusions cannot be drawn from the study (see Table 3). Subramaniam et al. (2018) later replicated these findings, highlighting that patients with schizophrenia had greater latency and reduced velocity gain of prosaccades in comparison to controls (see Table 3), this result reaching statistical significance. A study analysing eye movements in patients with schizophrenia in a natural, non-laboratory environment (Dowiasch et al. 2016) also identified impaired saccades. Self-initiated saccades to pre-defined targets were associated with significant ‘under-shoots’ in patients with schizophrenia (see Table 3); however, during a ‘free-viewing’ task, saccade amplitude and peak velocity measurements were similar to that of controls. It is unclear how much influence testing conditions have upon saccades; however, there still appears to be some saccadic abnormality found consistently within this patient subgroup.

#### Vergence

There is a no clear consensus regarding vergence eye movements in patients with schizophrenia, and further research is required to expand this evidence base. Bolding et al. (2012) found that the near point of convergence for patients with schizophrenia was comparable to that of controls (see Table 3); however, when surveyed, patients were more likely to subjectively report asthenopic symptoms similar to those experienced in convergence insufficiency.Additionally, patients with schizophrenia were more likely to fail Sheard’s criteria, which states that patients should have fusional reserves of at least twice the size of their heterophoria (Sheard 1938). Patients with schizophrenia may therefore experience more difficulty controlling heterophorias, this strain causing symptoms of asthenopia. However, the relatively small sample size (see Table 3) and lack of further research supporting Bolding et al.’s (2012) conclusions calls into question the clinical significance of this finding, highlighting a need for additional research before conclusions can be drawn. Additionally, the ‘convergence insufficiency symptom survey’ used by Bolding et al. (2012) was open to response bias from both patients and controls, undermining the reliability of this data.

Lab-based, case-control studies provide a consensus that patients with schizophrenia have a high incidence of eye movement abnormalities, such as reduced gain and catch-up saccades during smooth pursuit (Friedman et al. 1992; Hutton et al. 1995), and increased latency of prosaccades (Sereno & Holzman 1995; Subramaniam et al. 2018) in comparison to controls. Dowiasch et al. (2016) question how applicable this is to real-life viewing conditions, and the current evidence base provides little understanding of the relationship between eye movement abnormalities and daily functioning. Morita et al. (2018) highlighted a correlation between increased severity of eye movement abnormality and decreased hours spent in work for patients with schizophrenia. However, conclusions cannot be drawn as to how or why eye movement abnormalities impacted work hours, and this may have been a correlational relationship. The clinical characteristics of the vergence system also remain unclear, with no definitive conclusion emerging from the literature analysed, however it appears that these patients may suffer with asthenopic symptoms relating to fusional reserves (Bolding et al. 2012).

### Strabismus

The global prevalence of strabismus was estimated to be 1.93%, with exotropia occurring more commonly than esotropia (Hashemi et al. 2019). Strabismus prevalence is reported to increase with age, and whilst esotropia is more common in western countries, exotropia is more common throughout Asia (Hashemi et al. 2019). Observational case-control studies have reported higher rates of strabismus amongst patients with schizophrenia than amongst healthy controls (Toyota et al. 2004; Yoshitsugu et al. 2006). Those with strabismus and schizophrenia were most likely to exhibit an exotropia (intermittent or constant) (Yoshitsugu et al. 2006). This is unsurprising, as exotropia is the most common strabismus sub-type in Asia (Hashemi et al. 2019), where these studies were conducted. However, the rates of exotropia found were significantly higher than general rates amongst Asian populations, and the odds ratio for constant exotropia in schizophrenia in Toyoto et al.’s study (2004) was highly significant; 20.6 (95% confidence interval, 5.03–56.2), highlighting a marked association between the two conditions within this cohort.

The findings of Shoham et al. (2021) contradict the theory that schizophrenia was associated with strabismus. Their longitudinal cohort study (see Table 2) focused on visual acuity; however, they suggested childhood strabismus did not contribute to an increased likelihood of ‘psychosis’ in later life. Furthermore, whilst Schiffman et al.’s 2006 longitudinal cohort study (see Table 4) attempted to highlight a link between schizophrenia and strabismus, the ‘trend’ identified did not reach statistical significance, despite the large cohort of participants. Further work is therefore required to determine whether childhood strabismus increases a patient’s likelihood of a schizophrenia diagnosis in later life. A broader cohort of participants could be investigated, as only Danish children born in Copenhagen were included in this study (Schiffman et al. 2006). Additionally, Shoham et al. (2021) only followed up with patients at ages 17 and 24 years, which may have been prior to illness onset. Peak incidence of psychosis is 25–29 years for women and 20–24 years for men (Kirkbride et al. 2006). These discrepancies therefore somewhat undermine the conclusions made regarding the association between schizophrenia and strabismus, which contrasts with the findings of Toyota et al. (2004) and Yoshitsugu et al. (2006).

Patients with strabismus are known to suffer with psychosocial problems, including decreased job prospects and difficulty with social interaction (Durnian, Noonan & Marsh 2011). In patients with schizophrenia and strabismus, negative symptoms such as social withdrawal could be exacerbated. Additionally, the visual symptoms of strabismus, such as diplopia (in decompensated or late onset squints), may be camouflaged by the positive symptoms of schizophrenia; this emphasises the importance of awareness of orthoptic conditions for health care clinicians, to ensure these patients can access appropriate care.

Toyota et al.’s (2004) and Yoshitsugu et al.’s (2006) findings (increased incidence of strabismus amongst those with schizophrenia) may lack external validity as they only examine the prevalence of strabismus within a central Japanese population. Goseki and Ishikawa (2017) found higher rates of exotropia than esotropia within a cohort of 1,214 Japanese adults (a ratio of 18.5:1, respectively), and 3.6% of all participants demonstrated intermittent or manifest strabismus. As previously discussed, Hashemi et al. (2019) suggested the global prevalence of strabismus was 1.93%; this comparison therefore highlights a greater incidence of strabismus within a Japanese population than is considered average globally. Whilst the rates of strabismus found in patients with schizophrenia in both cohorts was undeniably significant, further research is needed to examine strabismus incidence in schizophrenia across a wider variety of ethnicities. Furthermore, the participants with schizophrenia originally included in Toyota et al.’s (2004) study were also recruited for Yoshitsugu et al.’s (2006) study, meaning that the findings of these studies only represent a limited range of individuals, rather than corroborating the findings across a wider group of participants.

The validity of these findings should also be scrutinised due to the lack of standardised testing methods employed. Yoshitsugu et al. (2006) used one ‘medically trained’ doctor to perform an ophthalmological assessment for all participants; only 16 out of the total 475 patients with schizophrenia were ‘cross-examined’ by an ophthalmologist. Consequently, this study has questionable inter-observer reliability, and the ophthalmological assessment of strabismus may not have been entirely accurate. Whilst findings were confirmed by an ophthalmologist in Toyota et al.’s study (2004), their methodology left a multitude of questions unanswered; they do not provide details regarding the size of deviations, fixation distances for the cover uncover and alternate cover tests, or whether patients with manifest strabismus experienced diplopia. This therefore means we are unable to distinguish the specific type of exotropia or esotropia this cohort presented with, which may be clinically relevant. Additionally, Schiffman et al. (2006) did not use an eye care clinician to perform the ‘eye examination’ for their cohort, instead using a neurologist. Subsequently, they do not provide information regarding the size or control of deviations noted. It is also unclear whether the cover test was performed in multiple positions of gaze, potentially disregarding the presence of incomitant deviations. These methodological inaccuracies undermine the validity and clinical relevance of these studies.

The exact relationship between strabismus and schizophrenia remains uncertain. Whilst there appears to be evidence highlighting a significantly high incidence of strabismus in this patient subgroup (Toyto et al. 2004; Yoshitsugu et al. 2006), this finding has not yet been corroborated across a range of ethnicities, and the incidence of diplopia within this subgroup is unknown. Furthermore, longitudinal cohort studies have not found an association between strabismus in childhood and incidence of schizophrenia diagnosis in later life (Schiffman et al. 2006; Shoham et al. 2021).

Certainly, those working within mental health services should be aware that strabismus, in particular exotropia, may be more frequently seen in patients with schizophrenia, in order to signpost symptomatic patients to orthoptic services and ensure continuity of care.

### Stereopsis

Stereoacuity in patients with schizophrenia was not extensively reported; however, some studies suggest it may be reduced in comparison to that of ‘healthy’ controls. Schechter et al. (2006) found that patients with schizophrenia had reduced stereoacuity (see Table 5) compared to controls, tested using the Graded Circles Stereotest (frequently used in American and Canadian Ophthalmology practice (Vancleef & Read 2019)). This finding was later confirmed by Hui et al. (2017), who found that patients with schizophrenia had statistically lower stereoacuity scores than controls on the Titmus Stereo test (see Table 5), within a cohort of Han Chinese participants. These studies suggest a potential relationship between schizophrenia and reduced stereoacuity, perhaps relating to altered visual processing or sensory perception.

Barbato et al. (2014) found no significant difference in stereoacuity level between young adults at high clinical risk of psychosis and controls (see Table 5). Clinical risk was assessed using the Structured interview for Prodromal Syndromes, which attempts to identify individuals who may go on to develop psychosis. These findings could imply that the onset of reduced stereoacuity in patients with schizophrenia, as observed by Schecter et al. (2006) and Hui et al. (2017), does not precede clinical symptoms. However, longitudinal data is required in order to understand whether these patients later developed schizophrenia and, if so, whether stereopsis deteriorated following onset. These findings may have been influenced by the artificial viewing conditions of lab-based studies, which are not always reflective of real-world viewing. When the Graded Circles Stereotest was used in a cohort of children (mean age 8 years), Adler, Scally and Barrett (2012) found that the majority of participants improved by ‘one’ testing level on repeated testing, with familiarity aiding performance. Whilst it is unclear whether this data can be extrapolated to reflect that of adult patients, it is worthwhile considering whether familiarity with stereoacuity testing may have impacted the discrepancy in scores found between patients and controls. Additionally, only medicated patients were included in Schechter et al.’s (2006) and Hui et al.’s (2017) studies, and their findings may therefore be reflective of antipsychotic medication effects on stereopsis, rather than schizophrenia specifically.

Although the findings of Schecter et al. (2006) and Hui et al. (2017) reached statistical significance, the clinical significance of these studies should be considered. Morris et al. (2005) highlight that monocular individuals are more likely to struggle with visual-motor tasks and reading in comparison to those with BSV, however patients with schizophrenia in both studies retained binocularity with only a slight disparity from that of controls. Normal limits of stereoacuity are variable, and clinical stereotests often do not accurately measure the threshold of stereoacuity, designed instead to identify degree of impairment (Read 2015). Additionally, both studies found no correlation between clinical symptoms of schizophrenia and stereoacuity level. These considerations challenge the clinical significance of these findings, especially as there is no prescribed orthoptic intervention for reduced stereoacuity when the cause is unidentifiable.

## Future research

The review examined a diverse range of sources, yet very little data specific to orthoptics and orthoptic assessment was yielded, highlighting a gap in the current evidence. A further limitation was the lack of ethnic or geographical diversity in the cohorts reported, limiting the generalisability of the findings to other populations. There was a limited range of literature exploring the possible associations that have been suggested between strabismus, reduced stereopsis, anisometropia or amblyopia and schizophrenia.

The effect of antipsychotic medication on smooth pursuit has been investigated (Friedman et al. 1992; Hutton et al. 2001); however, further research is required to understand the effects of medication upon the saccadic and vergence systems of patients with schizophrenia. Additionally, further research to investigate whether the structural ocular changes reported by Cumurcu et al. (2015) are present before commencing antipsychotic drug therapy would allow greater understanding of whether changes are related to schizophrenia or the antipsychotic treatments. Further study of how structural ocular changes affect visual function is warranted. However, ethical implications of using randomised control trials within this subgroup, especially relating to medication effects, limits the scope to which findings can be separated between ‘medicated’ and ‘unmedicated’ cohorts.

Future research examining how eye movement abnormalities, strabismus or other orthoptic findings may influence the symptoms of schizophrenia and daily functioning would be beneficial. Zheng et al. (2015) reported reduced visual acuity in patients with schizophrenia was related to lower quality of life scores and Cumurcu et al. (2015) related astigmatism to negative symptoms of schizophrenia. Strengthening this evidence may allow professionals to target orthoptic assessment and intervention to those considered most vulnerable to schizophrenia symptoms and worse daily functioning.

## Conclusion

In light of the evidence reviewed, orthoptists and eye care professionals should be aware that there was a higher incidence of ocular abnormalities in patients with schizophrenia compared to healthy controls. Orthoptic conditions such as strabismus (in particular exotropia) and eye movement abnormalities (reduced smooth pursuit gain and increased prosaccadic latency) appear to have a higher incidence amongst this subgroup. A high prevalence of reduced visual acuity and increased refractive error have also been identified. The current evidence base also suggests a relationship between some of these ocular abnormalities and negative symptoms of schizophrenia; however, further research is required to fully understand the extent to which orthoptic conditions influence schizophrenia, the symptoms of schizophrenia, and daily functioning for these patients.
